# Factors influencing college students' online rumor refuting behavior during major public health crises: the moderating effect of group norms

**DOI:** 10.3389/fpsyg.2024.1412034

**Published:** 2024-06-26

**Authors:** Hongmei Xia, Yu Liu, Guanghui Hou

**Affiliations:** ^1^College of Public Administration and Law, Hunan Agricultural University, Changsha, China; ^2^College of Law, Shantou University, Shantou, China

**Keywords:** rational behavior theory, information seeking, group norms, rumor refuting, stimulus-organism-response theoretical framework

## Abstract

This study integrates SOR (Stimuli-Organism-Response) theoretical framework and rational behavior theory within a theoretical framework, incorporating group norms as a moderating factor to investigate the psychological mechanisms influencing Chinese college students' online rumor-refutation behavior amidst public health crises. Using the structural equation modeling research method, data was collected via questionnaires from 1,254 participants in the context of the COVID-19 pandemic. The findings indicate that both online and offline information seeking are positively correlated with college students' attitudes and subjective norms. Moreover, the attitudes and subjective norms of college students are positively correlated with the online rumor refuting behavior. Furthermore, group norms serve to strengthen the connection between college students' attitudes and their engagement in online refuting rumors. These results illuminate the psychological underpinnings driving college students' online rumor-refuting actions, offering practical and policy implications for effectively managing rumor behaviors.

## 1 Introduction

In 2020, the COVID-19 pandemic emerged as the most significant public health crisis confronting humanity in a century (Guitton, 2020). A multitude of rumors and misinformation concerning the causes, prevention, and treatment of the disease proliferated across social media platforms (Tasnim et al., 2020). Promptly recognizing the significance of these rumors, the WHO identified this surge as the first global “infodemic” (Cinelli et al., 2020; Zarocostas, 2020). In other words, the outbreak of a public health crisis often triggers the circulation of rumors, resulting in physical and psychological harm to individuals. Particularly, the onset of a public health crisis can induce anxiety among college students regarding academic setbacks and worsen the toll on their mental wellbeing (Cao et al., 2020). The spread of misinformation can exacerbate the situation, so personal involvement in refuting rumors becomes crucial, especially for college students who are active on social media platforms.

In essence, social media has entrenched itself in people's daily lives as the primary mode of communication (Luo et al., 2023), especially amid social crises. It not only hastens the dissemination of rumors but also emerges as a crucial platform for debunking them (Tripathy et al., 2013). According to Alexander (2014) study, social media exhibits substantial potential in enhancing emergency response and management due to its ability to swiftly and extensively disseminate information more efficiently than traditional media during crises. Emergency management agencies frequently leverage social media platforms like Twitter and Facebook to enhance risk communication, allay public apprehensions, and alleviate the negative impacts of a crisis (Gui et al., 2017). Correspondingly, the public's utilization of social media has surged significantly, underscoring its pivotal role in crisis communication (Niles et al., 2019). In summary, social media has evolved into a critical tool for authorities and the general populace to combat online falsehoods. Concurrently, adolescents—especially college students—constitute the largest and rapidly expanding demographic cohort utilizing the internet for both informational and communicative purposes (Greenfield, 2004; Subrahmanyam and Lin, 2007). Consequently, exploring the factors influencing college students' engagement in debunking online rumors during public health crises is imperative.

Existing literature focuses mainly on the spread of COVID-19-related rumors on social media during public health crises, with less emphasis on online rumor rebuttals (Ding et al., 2020; Mourad et al., 2020). Past studies on online rumor dissemination have centered on the sharing processes and motivations, separately (Zhao et al., 2013, 2016; Lee et al., 2015; Kwon and Rao, 2017; Oh et al., 2018; Chen, 2019). For instance, Zhao et al. (2013) employed the SIR (susceptible, infected, and recovered) model to study rumor propagation. In terms of motivations, Zhao et al. (2016) examined social media during social crises, while Kwon and Rao (2017) explored government internet surveillance contexts. Luo et al. (2021) examined the correlation between peer status, peer communication, fear, personal rumor sharing, and the potential moderating impact of health self-efficacy. Furthermore, existing research on rumor-refutation primarily delves into the factors influencing individual rumor denial and the efficacy of debunking rumors. For instance, Liu et al. (2023) explored variables affecting both individual rumor refutation and propagation, while also considering the moderating influences of health self-efficacy and critical thinking. Key factors for effectively countering rumors include the source (Tang and Lai, 2018), content (Ruan and Xia, 2020) and communication channels (Huang, 2020) used in refuting them. Additionally, a subset of scholars has scrutinized various demographic cohorts. For example, Sun et al. (2020) analyzed the determinants of rumor re-circulation among middle-aged and elderly demographics during a public health crisis, suggesting a positive correlation between these populations' inclination to re-circulate rumors during the COVID-19 pandemic and their belief in rumors and personal anxiety levels, with negative associations with their ability to discern rumors and comprehend the consequences of rumor dissemination.

In summary, present literature primarily focuses on rumor propagation rather than refutation behavior. Earlier research on rumor conduct has predominantly concentrated on individual physical and mental states, such as health self-efficacy and critical thinking, with minimal consideration for the potential moderating influence of group norms from a sociological perspective. Moreover, the psychological underpinnings driving college students' online rumor-refutation behavior have been underexplored. Hence, this paper integrates the SOR theoretical framework and rational behavior theory, incorporating group norms as a moderating variable to investigate factors influencing college students' online rumor-rebutting actions. The study aims to achieve two main objectives: firstly, to develop an effective model to comprehensively understand the decision-making process of college students to refute rumors during major public health emergencies by merging stimuli-organism-response (SOR) theoretical framework and rational behavior theory. Secondly, this paper investigates the correlation between various factors and college students' online rumor-refutation behavior amidst significant public health crises. These factors encompass information sources, attitudes, subjective norms, and the moderating influence of group norms.

## 2 Theoretical grounding and hypothesis development

### 2.1 Stimuli-organism-response theoretical framework and and rational action theory

Stimuli-organism-response (SOR) theoretical framework was proposed by Woodworth (1929) as an extension to the classic theory of the stimulus-response model suggested by Pavlov (1927). The SOR theoretical framework proposes that certain environmental factors can impact individuals' emotional and cognitive state, leading to specific behavioral outcomes (Mehrabian and Russell, 1974). SOR theoretical framework is comprised of three constructs i.e., stimulus, organism, and response, which decide the behavioral outcome of an event (Skinner, 1935). Stimulus refers to environmental factors that affect an organism's state. Organisms are represented by cognitive and affective states that mediate the relationship between stimuli and individual responses. Response embodies the final results and behaviors exhibited by the stimulus receiver. The SOR theoretical framework has been useful in a variety of research areas in interpreting individual behavior (Kurniawan et al., 2021), and it has been successfully applied to examine human behavior during the COVID-19 pandemic (Song et al., 2021; Soroya et al., 2021), such as spreading rumors, refuting rumors, etc. In public health emergencies, human behavior can change due to environmental factors. Based on the SOR theoretical framework, Pal et al. (2020) studied the effect of message attributes on rumor refutation; Luo et al. (2021) explain how peer communication and peer status lead to fear of COVID-19 and thus promote rumor-sharing behavior; Liu et al. (2023) studied the impact of online and offline epidemic-related information and fear on personal rumor sharing and rumor refutation during the COVID-19 pandemic. Therefore, this study uses SOR theoretical framework as a visual framework to investigate college students' online rumor-refuting behavior.

In addition, the theory of rational action (TRA) states that an college students' decision is due to the will effort of a particular action (Fishbein, 1979; Han and Kim, 2010). The TRA has two components – the attitude and subjective norm. Attitude is defined as an individual's favorable or unfavorable assessment of a particular behavior. Subjective norm is defined as the effect of “perceived social pressure to perform or not perform the behavior.” In this study, attitude denotes the supportive stance of college students toward online rumor refuting amid significant public health crises. Subjective norm pertains to the social pressure felt by college students if they choose not to refute rumors online during the epidemic. Both these affect behavioral intention, which is a proxy for actual behavior itself (Ajzen, 1991; Eagly and Chaiken, 1993). Rational action theory has been widely used to explain individual behavior, and this theory has also been applied to the study of individual online rumor behavior (Pal et al., 2019). The attitude and subjective norm discussed in the theory of rational behavior pertain to college students' emotional attitude and cognition toward something.

Hence, this study aims to examine college students' behavior in refuting online rumors during significant public health emergencies, drawing from the SOR theoretical framework and rational behavior theory. In this context, the “stimulus” (S) encompasses various external sources of information that impact college students, both online and offline. These “stimuli” can influence the emotions and cognition of “organism” (college students), where attitude and subjective norms in the theory of rational behavior represent personal emotions and cognition. Therefore, in this paper, “organism” (O) represents the attitudes and subjective norms of college students. According to the theory of rational behavior, it is evident that “organism” (college students), influenced by attitudes and subjective norms, will impact personal volition and actions. As a result, in this study, the “reaction” (R) focuses on the behavior of college students when debunking online rumors.

### 2.2 Information seeking, attitude, and subjective norm

Information seeking refers to the process of obtaining information through certain technical means or methods for a specific purpose and within a certain scope (Agosto and Hughes-Hassell, 2005). Throughout the pandemic, individuals were inundated with vast amounts of COVID-19-related information from various online sources. By engaging in online information seeking, individuals acquired a deeper understanding of the virus, adopted precautionary measures, and reduced anxiety (Zheng et al., 2023). Consequently, information seeking, particularly through online seeking, can be seen as a coping mechanism for managing uncertainty following the onset of the initial COVID-19 outbreak (Tandoc Jr and Lee, 2022). During the fight against COVID-19, people's access to epidemic-related information can be categorized into two sources: online information from social media and offline information from traditional media (Guo et al., 2023). Rumor behavior during the COVID-19 pandemic has been studied by Liu et al. (2023) from the perspective of information processing, where information sources were divided into online and offline information search. In this paper, information seeking is categorized into online information search, which involves seeking epidemic-related information on platforms like Weibo, Twitter, and others; and offline information seeking, which includes discussions with friends, family, colleagues, or offline media exposure, to gather epidemic-related information (Wang et al., 2020).

Information seeking has the potential to facilitate changes in beliefs and behaviors (Etingen et al., 2013). Personal beliefs are influenced by attitudes and subjective norms, thus information seeking can impact an individual's attitudes and subjectivity. With the need for knowledge, more and more people, particularly college students, now rely on online platforms, including micro-blogs like Twitter, video sharing sites like YouTube, and Wikipedia, to get news and updates (Kim et al., 2014). Additionally, individuals will also turn to offline means by seeking information from people around them about the epidemic. Peer communication has been found to influence college students' attitude and behavior toward the epidemic (Luo et al., 2021). Liu et al. (2023) also noted that online and offline communication among people during the epidemic impacted their fear of the virus and their behavior. In other words, both online and offline information searches regarding the epidemic influence individuals' attitudes, subjective norms, and behaviors related to the epidemic.

Previous studies have indicated that different sources of information have a positive impact on individuals' emotions and cognition during the epidemic (Soroya et al., 2021). For example, one study found that different sources of information can elicit fear emotions in individuals (Liu et al., 2023). When college students actively seek information regarding the epidemic, whether online or offline, it signifies their desire for accurate information, fostering a more positive attitude toward online rumor-refutation. Additionally, as intellectual figures, college students are amidst individuals seeking precise information from them, thereby heightening expectations for them to counter online rumors. Hence, college students may perceive significant social pressure if they refrain from debunking online rumors, thereby experiencing notably strong subjective norms. In other words, different sources of information significantly influence individuals' attitudes, subjective norms and perceptions about the epidemic (Zheng et al., 2023). Based on the above analysis, this paper proposes the following hypotheses:

H1. Online information seeking is positively correlated with college students' positive attitude toward online rumor refuting behavior.
H2. Online information seeking is positively correlated with college students' subjective norm.
H3. Offline information seeking is positively correlated with college students' positive attitude toward online rumor refuting behavior.
H4. Offline information seeking is positively correlated with college students' subjective norm.

### 2.3 Attitude, subjective norm, and rumor refuting

Rumor refuting involves exposing the rumor by presenting evidence that supports the truth, and it is a good methods to curbing rumors (Wen, 2014). In other words, refuting rumors is a kind of rumor blocking strategy, which is to clarify, refute and correct rumors, and its fundamental purpose is to curb the spread of rumors. Studies have indicated that the behavior of rumor refutation is linked to various factors, including the quality and credibility of the response (Wang et al., 2022), speed of refuting rumors and response to rumors (Zhang et al., 2022), the utilitarian and hedonic values of the response (Pal et al., 2020), fear emotion (Liu et al., 2023) and individual characteristics (Liang et al., 2022). From the perspective of social psychology, the influence of emotional factors on rumor-refuting behavior is mainly explained by SOR theoretical framework. For example, Liu et al. (2023) studied rumor behavior during COVID-19 pandemic based on SOR theoretical framework, and the research conclusion showed that there was a moderate positive correlation between fear and rumor refuting. However, the above studies mainly focus on the organism's attitude (i.e., fear), but neglect the measurement of organism's cognition, resulting in the limitations of the study of emotional factors on rumor-refuting behavior. Therefore, this paper presents the theory of rational behavior as a means to assess college students' attitude and cognition toward a particular subject. According to this theory, college students' behavior is influenced by their attitudes and subjective norms. Zhao et al. (2016) researched how social media users engage in anti-rumor behavior during social crises. They integrated the theory of planned behavior and normative activation model to analyze this. Their findings indicate that attitude influences personal willingness, which in turn influences anti-rumor behavior. Key factors predicting actual anti-rumor actions include subjective norms, perceived behavioral control, and awareness of potential consequences. Building upon this analysis, the following hypotheses are proposed:

H5. Attitude is positively correlated with college students' online rumor refuting behavior.
H6. Subjective norm is positively correlated with college students' online rumor refuting behavior.

### 2.4 Group norm as the moderating role

According to social identity theory, individuals' sense of self is influenced by their membership in social groups or categories (Tajfel and Turner, 1979; Abrams and Hogg, 2006). Building on this theory, self-categorization theory suggests that when individuals define themselves in terms of a specific social group, or ingroup, they develop explicit or implicit guidelines for how ingroup members should think and behave, known as group norms (White et al., 2002). These group norms then influence individuals' behavior. Individuals tend to emphasize differences between ingroup and outgroup members, as well as similarities among ingroup members, on stereotypical dimensions, in order to solidify their identity as group members (Turner, 1985; Turner et al., 1987). As a result, individuals are more likely to engage in certain behaviors when there is normative support from their ingroup (Terry and Hogg, 1996).

With this in mind, this paper aims to examine the impact of group norms on individuals' behavior in regards to refuting rumors. Previous studies have consistently shown that group norms have a positive influence on individual behavior. For instance, Terry and Hogg (1996) found that the group norms established by friends and peers significantly predicted university students' intentions to engage in regular exercise, particularly for those who strongly identified with their ingroup. Similarly, Johnston and White (2003) found that the construct of group norms significantly predicted university students' intentions to engage in binge-drinking, regardless of the level of ingroup identification. Furthermore, Robinson et al. (2016) found a significant association between group norms and adolescents' use of social networking sites. Taken together, these findings highlight the effective role of group norms in regulating individual behavior. Based on the above analysis, this paper proposes the following hypotheses:

H7. Group norm will positively moderate (strengthen) the relationship between college students' attitudes and the behavior of online rumor refuting.
H8. Group norm will positively moderate (strengthen) the relationship between college students' subjective norm and the behavior of online rumor refuting.

## 3 Research methodology

### 3.1 Participants

The survey was conducted from 8 October to 30 December 2023. A combination of offline and online questionnaires was utilized to gather data. For the offline survey, the researchers randomly intercepted college students at the entrance of the university's teaching building and distributed questionnaires. In total, 500 questionnaires were gathered. For the online survey, Questionnaire Star (http://www.sojump.com/) was employed (Sun et al., 2017; Li et al., 2021). To maintain consistency in both online and offline data collection, the questionnaire begins with an initial screening question: “Are you currently a college student (junior college students, undergraduate, post-graduate)?” Participants who answer affirmatively continue with the following questions, while those who answer negatively exit the questionnaire. To encourage participation, respondents were offered a small cash. In total, 1,500 questionnaires were distributed both online and offline, resulting in 1,254 responses. Invalid questionnaires, which consisted of those where all answers were the same, some answers were missing, or answers were clearly contradictory, were excluded. Ultimately, 1,038 valid responses were considered for data analysis. The demographic data of the sample are displayed in Table 1, indicating that 46.82% of the respondents were male, 89.50% were aged 18-25, 50.96% possessed a bachelor's degree or higher, and merely 1.60% lived alone.

**Table 1:** Sample demographics.

**Characteristics**	**Levels**	**Frequency**	**Percentage (%)**
Gender	Male	486	46.82
	Female	552	53.18
Age group	< 18	87	8.38
	18-25	929	89.50
	26-30	13	1.25
	>30	9	0.87
Education	Junior college students	509	49.04
	Undergraduate	388	37.38
	Postgraduate	141	13.58
Living condition	Live alone	17	1.60
	Live with roommates	948	91.30
	Live with relatives	70	6.70
	Live with others	3	0.30

### 3.2 Measures

The questionnaire consisted of two parts: demographic information and constructs relevant to the study. The first part collected participants' gender, age group, education, and living conditions. The second part focused on the constructs and utilized a 7-point Likert scale, ranging from 1 (strongly disagree) to 7 (strongly agree). To ensure content validity, we employed the double-translation/back-translation method to ensure consistency between the Chinese and English versions. Additionally, we conducted a pilot study with 70 individuals to revise the questionnaire. The measures for all constructs were adapted from previous literature to suit the context of our study.

#### 3.2.1 Online information seeking

We evaluated participants' tendency to seek virus-related information online using a three-item scale adapted from Oh H. J. et al. (2013). This scale measured their preference for using online social media platforms to obtain such information. For example, one item stated, “During the epidemic, I frequently rely on social media platforms like Weibo to access virus-related information.”

#### 3.2.2 Offline information seeking

To assess participants' offline information seeking habits, three items adapted from Suki and Suki (2019) were utilized. The projects evaluated how often participants interacted face-to-face to discuss virus-related topics with friends, roommates, and relatives on a daily basis, and their preference for obtaining information through traditional media. An example of the item used reads as follows: “I frequently engage in offline discussions about virus-related information with friends, roommates, or relatives during the epidemic.”

#### 3.2.3 Attitude

We assessed participants' attitude using three items modified from Han et al. (2010). These items gauge college students' perspective on pandemic information. For example, one item stated, “I believe that engaging in online debunking of rumors is beneficial during the epidemic.”

#### 3.2.4 Subjective norm

We evaluated participants' subjective norm by employing a three-item scale derived from Han et al. (2010) and Chen and Tung (2014). These items gauged the college students' perception of social pressure regarding their involvement in online rumor refutation during the epidemic, such as “I believe that the majority of individuals who are significant to me are supportive of my participation in combating rumors online during the outbreak.”

#### 3.2.5 Group norm

The measurement of group norm consisted of three items adapted from Terry and Hogg (1996), White et al. (2008), and Robinson et al. (2016). These items assessed the impact of social identity and self-classification of individuals' peers on their behavior. For example, one item stated, “Many of my friends have actively participated in debunking rumors during the pandemic.”

#### 3.2.6 Rumor refuting

The measurement of rumor refuting involved three adapted items from Venkatesh et al. (2003), Oh O. et al. (2013), and Zhang et al. (2021a), which assessed participants' actions in debunking rumors. These items specifically examined behaviors like sharing refutations of virus-related rumors on social media. For example, one item stated, “I have shared rumor-dispelling information about the epidemic on social media (Weibo/wechat, etc.) during the epidemic period.”

#### 3.2.7 Covariates

Covariates refer to variables that can potentially influence study outcomes, aside from the independent variables. Previous studies have indicated that demographic factors, including age, gender, education, and living conditions, may impact individuals' perceptions and behaviors (Luo et al., 2021; Ma et al., 2022). Therefore, we included these variables as covariates in our research model.

## 4 Data analysis

The data were analyzed using SPSS 27 and AMOS 26. To adhere to the guidelines provided by Anderson and Gerbing (1988), a two-step model was employed. Firstly, a measurement model was estimated through confirmatory factor analysis (CFA) to assess the reliability and validity of the items and constructs. Secondly, structural equation modeling (SEM) was utilized to evaluate the model fit and test the hypotheses.

### 4.1 Measurement model

We assessed the reliability and validity of the constructs using the AMOS software before conducting the regression analysis. To evaluate the construct validity, confirmatory factor analysis was employed, following Marsh et al. (2004) guidelines. The obtained results indicated that the model fit the data reasonably well (χ^2^/df = 3.431; CFI = 0.978, TLI = 0.973; RMSEA = 0.048; PNFI = 0.761). Initially, we examined the convergent validity, with the findings displayed in Table 2. It is evident that all the items' loadings were above 0.7, meeting the criterion for convergent validity. Furthermore, the Cronbach's alpha values exceeded 0.7, indicating high internal consistency. The composite reliabilities (CR) for all constructs were also above 0.7, supporting their reliability. Additionally, the average variance extracted (AVE) values were higher than 0.6, further confirming the constructs' convergent validity, following previous research by Fornell and Larcker (1981) and Chin and Marcoulides (1998).

**Table 2:** Reliability and validity.

	**Items**	**Factor loading**	**Cronbach's alpha**	**CR**	**AVE**
Online information seeking(ONI)	ONI1	0.862	0.854	0.871	0.695
	ONI2	0.893			
	ONI3	0.737			
Offline information seeking(OFFI)	OFFI1	0.810	0.861	0.870	0.690
	OFFI2	0.827			
	OFFI3	0.854			
Attitude(ATT)	ATT1	0.869	0.894	0.881	0.712
	ATT2	0.809			
	ATT3	0.853			
Subjective norm(SN)	SN1	0.888	0.950	0.916	0.785
	SN2	0.904			
	SN3	0.865			
Group norm(GN)	GN1	0.867	0.892	0.895	0.740
	GN2	0.900			
	GN3	0.811			
Rumor refuting (RR)	RR1	0.858	0.892	0.886	0.722
	RR2	0.850			
	RR3	0.841			

ONI, Online information seeking; OFFI, Offline information seeking; ATT, Attitude; SN, Subjective norm; GN, Group norm; RR, Refute rumors.

Moreover, the discriminant validities of the constructs are presented in Table 3. This is done by comparing the square root of the Average Variance Extracted (AVE) of each construct with the correlation coefficients pertaining to that construct. It is evident from the table that the square roots of the AVE values for all constructs are greater than the correlation coefficients with other constructs in the model. These findings attest to the satisfactory level of discriminant validity.

**Table 3:** Correlations and discriminant validity.

	**ONI**	**OFFI**	**ATT**	**SN**	**GN**	**RR**
ONI	**0.834**					
OFFI	0.605^***^	**0.831**				
ATT	0.304^***^	0.349^***^	**0.844**			
SN	0.259^***^	0.318^***^	0.553^***^	**0.886**		
GN	0.209^***^	0.295^***^	0.361^***^	0.359^***^	**0.860**	
RR	0.183^***^	0.228^***^	0.451^***^	0.452^***^	0.530^***^	**0.850**

The bold diagonal values in italics represent the square root of AVE; ^*^p < 0.05, ^**^p < 0.01, ^***^p < 0.001.

Next, to investigate the issue of multicollinearity, we utilized the variance inflation factor (VIF). The findings of our study revealed that the VIF values for all dimensions ranged from 1.212 to 1.534, with a tolerance >0.1. These results suggest that multicollinearity was not a significant concern based on the existing literature (Cohen et al., 2013; Zhang et al., 2021b).

Finally, we utilized Harman's single-factor test to determine the presence of common method bias (CMB). By performing exploratory factor analysis on all the data items using the no-rotation method, we aimed to identify the loading of the first factor among the extracted factors. If the test result is below 50%, it indicates that CMB is within an acceptable range. In our study, the loading of the first factor was calculated to be 37.502%, which falls below the recommended threshold of 50% (Podsakoff et al., 2003, 2012). Therefore, we can conclude that CMB is not a concern in this study.

### 4.2 Structural model

The goodness-of-fit indices of the theoretical framework were assessed using the structural model. However, the value of χ^2^/df in the fitting result of the structural model is too high. To address this, the model was modified by establishing a correlation between the attitude residual and the subjective norm residual. As a result, the modified model exhibits a good fit, as indicated by the following indices: χ^2^/df = 3.426 (< 5), CFI = 0.982 (>0.9), TLI = 0.977 (>0.9), RMSEA = 0.048 (< 0.08), and PNFI = 0.762 (>0.5).

**Figure 1:**
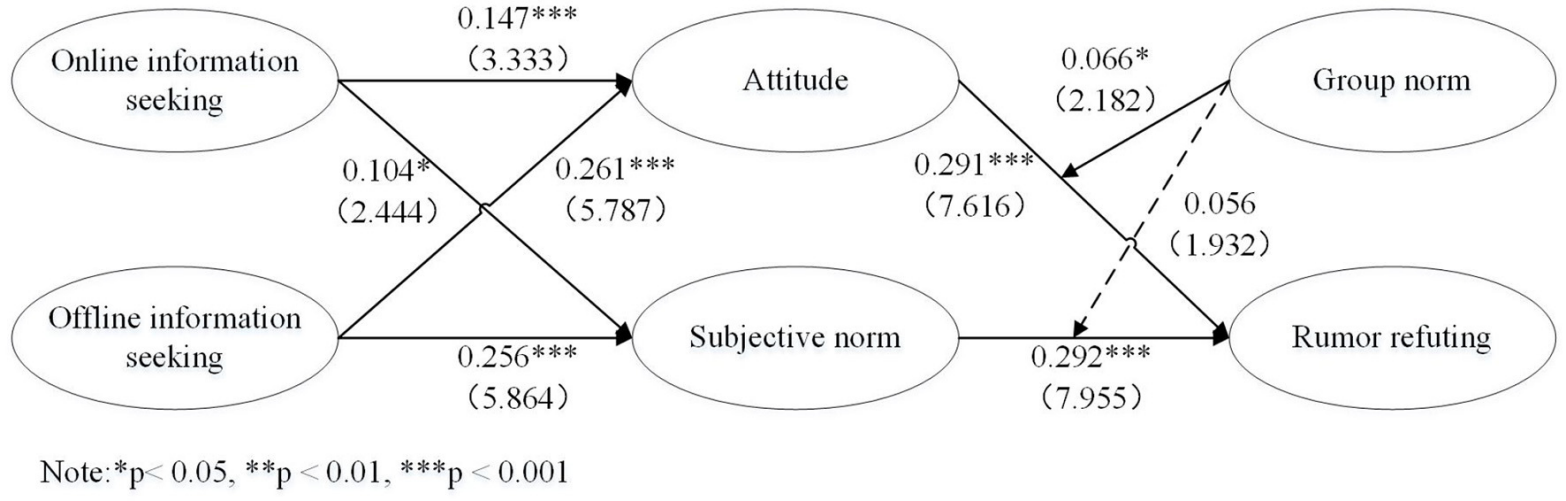
Path coefficient (standard error) in structural equation model.

Table 4 and Figure 1 presents the regression results for the hypotheses testing. The regression pathways from online information seeking and offline information seeking to attitude were found to be significantly positive, supporting H1 and H3. Similarly, the regression pathways from online information seeking and offline information seeking to subjective norm were also significantly positive, supporting H2 and H4. Additionally, the regression pathways from attitude and subjective norm to refuting rumors were found to be significantly positive, supporting H5 and H6. Finally, the regression pathway from interaction term 1 (attitude × group norm) to refute rumors was significantly positive, supporting H7. However, the regression pathway from interaction term 2 (subjective norm × group norm) to refute rumors was not significantly positive, indicating non-support for H8.

**Table 4:** Results of hypothesis testing.

**Hypothesized paths**	**Standardized beta coefficients**	***T*-Value**	**Result**
Online information seeking → Attitude	0.147^***^	3.333	Supported
Online information seeking → Subjective norm	0.104^*^	2.444	Supported
Offline information seeking → Attitude	0.261^***^	5.787	Supported
Offline information seeking → Subjective norm	0.256^***^	5.864	Supported
Attitude → Rumor refuting	0.291^***^	7.616	Supported
Subjective norm → Rumor refuting	0.292^***^	7.955	Supported
Attitude × Group norm → Rumor refuting	0.066^*^	2.182	Supported
Subjective norm × Group norm → Rumor refuting	0.056	1.932	Not supported

^*^p < 0.05, ^**^p < 0.01, ^***^p < 0.001.

### 4.3 Moderating effect of group norm

The moderating effects were assessed by calculating the mean-centered indicator values before multiplying the moderator variable with the predictor variables, which are also known as interaction terms. This method was validated by Ping (1995) as a suitable approach to evaluate path models involving interactions among latent variables. We calculated the effect size of the interaction effect to further assess the moderating role of group norms. Table 4 and Figure 1 presents the results of the path estimates and *t-*values for the interaction effect. Specifically, the moderating effect of group norms on attitude and refuting rumors is significant (*p* < 0.05), but the moderating effect on subjective norms and refuting rumors is not significant. Therefore, the research findings support H7 and do not support H8.

**Figure 2:**
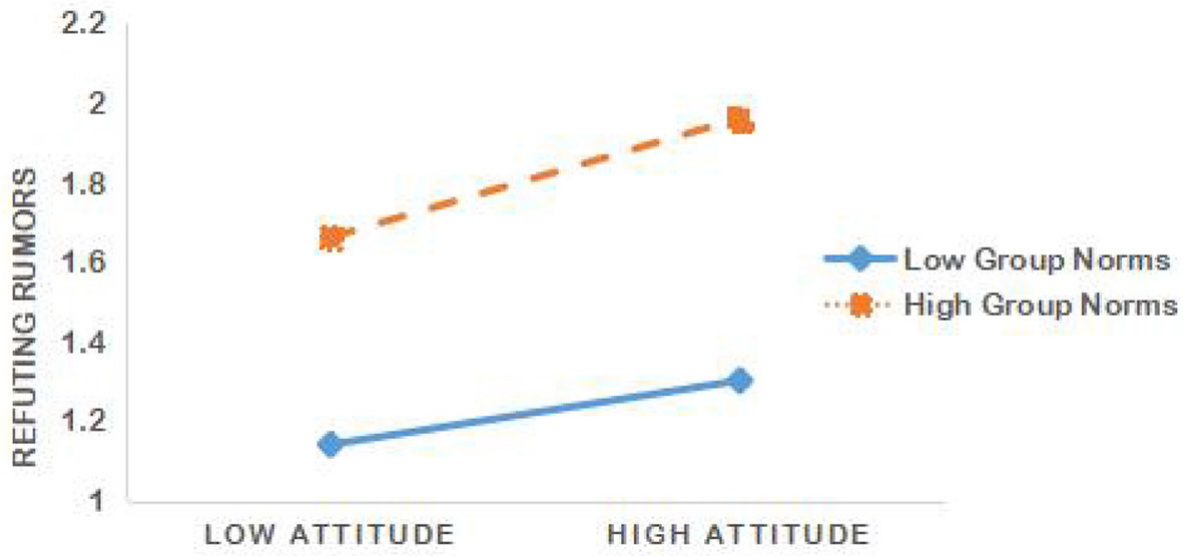
Moderation effect of group norms on the relationship between attitude and refuting rumors.

To further validate the moderating role of group norms (H7), we conducted a slopes test proposed by Aiken et al. (1991) to examine the interaction effect. We represented the moderator scores at high and low levels. As shown in Figure 2, the association between attitude and refuting rumors is stronger when individuals have high levels of group norms (dotted line) compared to low levels (solid line). This suggests that group norms strengthen the relationship between attitude and refuting rumors, supporting H7.

## 5 Discussion

### 5.1 Key findings

This study explores college students' behaviors in refuting online rumors during public health crises, using the SOR theoretical framework and rational behavior theory. The study yielded some significant findings.

First, the study confirmed the stimulus-organism response hypothesis. The results show that both online and offline information seeking are positively correlated with college students' attitudes and subjective norms. Different information sources show a positive correlation with individuals' attitudes, consistent with a prior study by Liu et al. (2023) that emphasized how both online information seeking and offline peer interactions can trigger feelings of fear in individuals. Different information sources also influence individuals' subjective norms, with online searches through social media, government channels, offline communication with peers or teachers, and information from traditional media all shaping expectations regarding rumor-refuting behavior. Therefore, both online and offline information seeking contribute positively to personal subjective norms. The study also revealed a strong positive correlation between online and offline information seeking and attitudes, with coefficients of (β = 0.147, *p* < 0.001) and (β = 0.261, *p* < 0.001) respectively, as well as subjective norms with coefficients of (β = 0.104, *p* < 0.05) and (β = 0.256, *p* < 0.001) respectively. Offline information seeking holds more sway over individual attitudes and subjective norms than online sources, given the prevalence of peer communication, traditional media, and offline channels like school propaganda. Even though social media is widely used, traditional media remains a crucial and trustworthy source during unforeseen events for reliable information (Wang and Zhuang, 2018). The expanding roles of traditional media in China, alongside offline societal interactions, still significantly impact personal information decisions, particularly within the Chinese context (Huang and Lu, 2017). Notably, information obtained through school bulletin boards, teacher-student exchanges, and offline peer interactions greatly influences college students' attitudes and subjective norms concerning the epidemic. This study concurs with Liu et al. (2023) on the positive link between offline peer communication, personal fear, and rumor-refuting behavior.

Second, the study found support for the organism response hypothesis. The empirical results show that the attitudes and subjective norms of college students are positively correlated with the online rumor refuting behavior. This finding aligns with the behavioral theory suggesting that individual attitudes and subjective norms influence one's intentions, thus reflecting in their actual behaviors. Specifically, personal attitudes positively influence rumor-refuting actions, consistent with prior research indicating that during social crises, personal attitudes impact individuals' intentions to combat rumors on social media, consequently leading to actual refutation behaviors (Zhao et al., 2016). In contrast, subjective norms also play a positive role in encouraging rumor-refuting actions, deviating from some previous study conclusions. For instance, Ajzen (1991) highlighted the varying impact of subjective norms on behavioral intent, indicating that personal attitudes and perceived control hold more sway over intentions than others' social pressures. Meanwhile, Terry and Hogg (1996) critiqued the inadequate conceptualization and assessment of subjective norm structures, emphasizing the underestimation of perceived social influences on behavior. However, Fishbein and Ajzen (1975) argued that the predictive power of subjective norms on intentions differs across populations and behaviors. In this context, for college students, subjective norms predominantly stem from expectations set by peers, friends, and teachers regarding rumor-dispelling behaviors. College students in China, who often live with roommates and within close-knit social circles, place significant value on others' opinions. Moreover, the strong student-teacher relationships in Chinese educational institutions, where teachers serve as mentors and guides, contribute to students' consideration of teachers' expectations toward them.

Finally, the study's findings highlight the significant role that group norms play in moderating relationships between variables. Specifically, group norms enhance the connection between college students' attitudes and their engagement in refuting rumors. Group norms heavily influence individual behavior regarding rumor refutation. Unlike subjective norms, which consider perceived pressure from significant others to act in a certain way, group norms encompass descriptive norms (such as perceptions of what important others actually do) and group attitudes (i.e., perceived attitudes of group members). Previous research has consistently demonstrated the predictive power of group norms on behaviors. For instance, Johnston and White (2003) discovered that group norms significantly enhanced the prediction of university students' intentions to binge-drink. Similarly, Mason and White (2008) noted that incorporating group norms related to friends and peers improved the accuracy of predicting young women's intentions to engage in frequent breast self-examinations. Moreover, Robinson et al. (2016) observed that group norms effectively predicted sun protection decisions among young female beachgoers in Australia. This study introduces group norms into the examination of college students' online rumor-refutation behaviors, an aspect often overlooked in prior research during major public health crises. College students, like other individuals, are susceptible to the influence of group norms. When the groups they are part of exhibit specific behaviors, their attitudes toward those actions are influenced, leading to the adoption of similar behaviors. This phenomenon is largely driven by the fear of social isolation among college students. Thus, group norms play a crucial role in regulating the relationship between college students' attitudes and their behavior in refuting rumors. Furthermore, while past studies typically focused on either group norms or subjective norms as singular influencers on individual behavior, this paper delves into the combined impact of group norms and subjective norms on college students' online rumor-refuting conduct. However, the moderating impact of group norms on the relationship between subjective norms and rumor refutation is not significant. One possible explanation is that subjective norms directly influence college students' behavior in refuting rumors. In the context of college students' response to rumors, the expectations of classmates, friends, and teachers regarding rumor refutation often align with their actual behaviors, thereby weakening the regulatory influence of group norms on the interplay between subjective norms and rumor refutation.

### 5.2 Theoretical implications

First, drawing on the SOR theoretical framework and rational behavior theory, this study advances a more comprehensive theoretical framework for understanding rumor-refutation behavior. This new model not only advances the existing SOR theoretical framework but also contributes to the literature on rumor refutation. While the SOR theoretical framework has been commonly utilized in exploring individual behavior amidst public health crises, such as rumor propagation and refutation (Pal et al., 2020; Luo et al., 2021; Liu et al., 2023), previous research primarily focused on factors like fear and belief in rumors when measuring organisms (Guo et al., 2023; Liu et al., 2023), with limited attention given to college students. By integrating the SOR theoretical framework with rational behavior theory, this study presents a novel theoretical framework for online rumor-refuting behavior that offers deeper insights into the dynamics of rumor refutation. Hence, this research not only enhances the SOR theoretical framework but also provides a more comprehensive understanding of online rumor-refuting behavior.

Second, this research sheds light on the impact of group norms on college students' online rumor-refuting behavior during public health crises. While group norms have been extensively studied in various research fields and proven to affect outcomes like individual behavior and performance (Baker and White, 2010; Robinson et al., 2016), their role in rumor-refuting behavior among college students in such crises remains underexplored. By incorporating group norms as a moderating factor in our research model, we discovered their significant enhancement of the positive correlation between attitude and online rumor-refuting behavior. Consequently, this study not only enriches the literature on online rumor-refutation behaviors but also underscores the crucial moderating impact of group norms in this context.

Third, this study offers a comprehensive understanding of college students' online rumor-refuting behaviors during public health crises, enriching existing literature on rumors. By examining information acquisition perspectives, it introduces a novel approach to studying rumor-refuting behaviors. Previous research primarily focused on online rumor propagation mechanisms, emphasizing social and traditional media, while overlooking the significance of offline communication in obtaining information (Xu, 2020; Liu et al., 2023). In this study, we explore how online social media, offline communication, and traditional media collectively impact rumor dispelling. Different channels may validate or debunk information from diverse sources. The findings indicate that offline information acquisition has a more substantial impact on college students' attitudes and norms regarding online rumor-refuting compared to online sources. Thus, emphasizing the simultaneous consideration of information retrieval from various outlets is crucial for effectively combating rumors.

### 5.3 Practical implications

On the one hand, in guiding college students' online rumor-refuting behavior, it is essential to consider the varying influences of different information sources. Firstly, educational institutions should facilitate access to offline information for students by providing dedicated spaces for offline information exchange and maintaining updated information on notice boards. Offline interactions with peers, friends, teachers, and other pertinent individuals during public health crises play a crucial role in shaping college students' attitudes toward dispelling rumors. Such offline engagements not only foster a positive outlook on rumor-refuting but also contribute to enhancing online refutation activities. Furthermore, vocalizing expectations for students to actively participate in online rumor-refuting during offline discussions can positively influence their subjective norms, consequently bolstering their online engagement in refuting rumors. Secondly, social media platforms need to rigorously verify the authenticity of content, swiftly address misinformation, and combat the spread of rumors. In times of public health crises, students frequently cross-reference online information with offline sources to ensure accuracy. Thirdly, policymakers should develop effective measures to regulate online rumors, enhance user awareness of cybersecurity, and reinforce online rumor monitoring mechanisms. For instance, authorities could conduct periodic workshops in schools to educate students about the propagation of online rumors, enhance their skills in discerning such misinformation, and motivate them to debunk rumors on digital platforms.

On the other hand, the school effectively plays the role of group norms and guides college students to participate in online refuting rumors. The behavior of a group has a considerable impact on the decisions made by its members, particularly within the college setting. Establishing and upholding group norms can robustly reinforce the connection between the attitudes of college students and their actions in dispelling rumors. Firstly, at the school level, fostering a positive group atmosphere and commending classes that actively engage in online rumor prevention can strengthen students' commitment. Schools can set examples, instill class pride, and encourage participation in anti-rumor activities. Secondly, on the teacher front, class educators can orchestrate group activities to bolster students' solidarity, direct their conduct, and promote responsible online behavior, including rumor prevention. Thirdly, at the class level, student leaders should lead by example, engaging in rumor prevention initiatives to inspire classmates and enhance class cohesiveness.

## 6 Conclusions and limitation

### 6.1 Conclusions

This study integrates SOR theoretical framework and rational behavior theory within a theoretical framework, incorporating group norms as a moderating factor to investigate the psychological mechanisms influencing Chinese college students' online rumor-refutation behavior amidst public health crises. The findings indicate that both online and offline information seeking are positively correlated with college students' attitudes and subjective norms. Offline information searches exhibit a stronger impact on these aspects compared to online searches. Furthermore, college students' attitudes and subjective norms regarding debunking rumors show a positive correlation with their online debunking behaviors. In addition, group norms serve to strengthen the connection between college students' attitudes and their engagement in online refuting rumors.

### 6.2 Limitation and future research directions

While this article provides valuable insights into debunking online rumors, it is important to acknowledge certain limitations. The study specifically focuses on Chinese college students, and rumor-refutation behaviors may differ in other cultural contexts. Moreover, the examination in this study is limited to attitudes and subjective norms as mediators, and group norms as a moderating factor. Therefore, future research could explore various areas. Firstly, there is a need to investigate online rumor-refutation behaviors across diverse demographic groups, including the elderly, and conduct comparative analyses on factors influencing these behaviors. Additionally, examining the online rumor-refutation behaviors of college students within different cultural contexts could yield valuable insights. Secondly, expanding the range of mediating variables to include beliefs in rumors and engagement on social media platforms could improve research outcomes. Finally, broadening the scope of regulating variables, such as critical thinking and self-efficacy, could enhance our understanding. Furthermore, conducting long-term follow-up studies to observe the evolving dynamics of group norms as a regulatory factor is recommended for future investigations.

## Data availability statement

The original contributions presented in the study are included in the article/supplementary material, further inquiries can be directed to the corresponding author.

## Ethics statement

Ethical review and approval was not required for the study of human participants in accordance with the local legislation and institutional requirements. Written informed consent from the patients/participants was not required to participate in this study in accordance with the national legislation and the institutional requirements.

## Author contributions

HX: Conceptualization, Methodology, Writing – review & editing, Data curation, Investigation, Writing – original draft. YL: Investigation, Supervision, Writing – review & editing. GH: Conceptualization, Methodology, Writing – review & editing, Supervision.
